# An Intelligent Sorting Method of Film in Cotton Combining Hyperspectral Imaging and the AlexNet-PCA Algorithm

**DOI:** 10.3390/s23167041

**Published:** 2023-08-09

**Authors:** Quang Li, Ling Zhao, Xin Yu, Zongbin Liu, Yiqing Zhang

**Affiliations:** College of Mechanical and Automotive Engineering, Liaocheng University, Liaocheng 252000, China; li18754773982@163.com (Q.L.); yx15553285576@163.com (X.Y.); liu_zongbin@163.com (Z.L.); yiqingzhang@lcu.edu.cn (Y.Z.)

**Keywords:** seed cotton, film, hyperspectral image, dimension reduction, convolutional neural network

## Abstract

Long-staple cotton from Xinjiang is renowned for its exceptional quality. However, it is susceptible to contamination with plastic film during mechanical picking. To address the issue of tricky removal of film in seed cotton, a technique based on hyperspectral images and AlexNet-PCA is proposed to identify the colorless and transparent film of the seed cotton. The method consists of black and white correction of hyperspectral images, dimensionality reduction of hyperspectral data, and training and testing of convolutional neural network (CNN) models. The key technique is to find the optimal way to reduce the dimensionality of the hyperspectral data, thus reducing the computational cost. The biggest innovation of the paper is the combination of CNNs and dimensionality reduction methods to achieve high-precision intelligent recognition of transparent plastic films. Experiments with three dimensionality reduction methods and three CNN architectures are conducted to seek the optimal model for plastic film recognition. The results demonstrate that AlexNet-PCA-12 achieves the highest recognition accuracy and cost performance in dimensionality reduction. In the practical application sorting tests, the method proposed in this paper achieved a 97.02% removal rate of plastic film, which provides a modern theoretical model and effective method for high-precision identification of heteropolymers in seed cotton.

## 1. Introduction

Cotton plays an irreplaceable part in the livelihood of the general population. Xinjiang is China’s largest major producer of long-staple cotton. However, due to the low rainfall and strong light, drip irrigation under the film is often adopted to boost yield, which is prone to mixing with impurities such as plastic film during mechanical picking. In the spinning and weaving processes, the residual film combined with seed cotton can result in a significant number of flaws, which can impact the strength and coloring effect of the yarn and lead to financial losses for the textile sector [1].

The existing mainstream cotton film removal processes include Mechanical separation, Electrostatic separation, and Optical color separation. Whitelock D. P. et al. investigated major impurity removal equipment in the US cotton industry. A rotating spiked cylinder was utilized to eliminate significant impurities from the seed cotton. These impurities were subsequently gathered in a separate box by means of a grid strip or screen. [2]. Zhang et al. used computational fluid dynamics (CFD) to model the electrostatic separation of mechanical cotton harvesting and residual plastic film by flying the experimental sample into an electric field at different speeds and applying different electric field forces [3]. With the increasing prevalence of machine vision, optical color separation has become a popular method for the intelligent classification of agricultural products. In a study conducted by Li et al., a machine vision system was utilized to gather information on the color, shape, and texture of foreign fibers in cotton. The resulting data were adopted to achieve a classification accuracy of 92.34% through multi-class support vector machine (MSVM) [4].

However, mechanical classification is challenging in the aspect of assuring accuracy and small-size film classification. Electrostatic separation becomes unstable for long-term work because of environmental conditions. Optical color selection relies on color and form characteristics, making it challenging to effectively classify film which is colorless, transparent, or irregularly shaped. Hence, it is imperative to investigate a dependable technique for identifying transparent films in seed cotton.

Hyperspectral imaging combines advanced knowledge from multiple disciplines to achieve a perfect fusion of traditional two-dimensional imaging techniques and spectroscopy. Guo and Ma described the linear relationship between spectra and data by the partial least squares (PLS) method, which could realize the analysis of adulterated rice and the prediction of pork meat fatty acids [5,6]. Zhang, Jiang, et al. employed the support vector machine (SVM) in combination with shortwave infrared hyperspectral techniques for cotton foreign matter classification, which significantly improved the detection rate of plastic films in cotton compared to conventional methods [7,8,9].

The above literature has yielded promising results. However, extracting features from hyperspectral images requires manual intervention and has limitations in feature mining. In addition, manual feature extraction of hyperspectral images requires considerable expertise and has subjectivity in feature mining and selection. Therefore, it is highly significative for hyperspectral image features to probe an automatic feature extraction method.

Deep learning is an advanced technology applied to image processing. It has the capability to automatically detect and analyze complex information, which helps to extract deeper features. The use of hyperspectral data greatly enhances the accuracy and efficiency of image recognition [10,11]. However, it is important to note that hyperspectral data can be affected by elevated latitudes and severe information redundancy issues. To efficiently extract feature information to support the training of deep learning models, data dimensionality reduction is commonly applied to improve the data processing speed [12]. Jia et al. employed a method for dimensionality reduction of hyperspectral images by flexible Gabor-based superpixel-level unsupervised linear discriminant analysis (LDA), which reduced a large amount of flexible Gabor (FG) features and increased the peculiarity of image features [13]. Kang et al. proposed a method based on PCA-EPFs for hyperspectral image (HSI) classification, which used principal component analysis (PCA) to reduce the dimension of the superimposing Edge-preserving features (EPFs). The literature not only represented the EPFs in the mean square sense but also highlighted the divisibility of pixels in EPFs [14]. To reduce the dimension of hyperspectral remote sensing images, Daniela Lupu et al. established an independent component analysis (ICA) method based on a stochastic higher-order Taylor approximation-based algorithm, which could identify local maxima and facilitate minibatching [15]. The previous researchers have utilized LDA, PCA, and ICA techniques for reducing the dimensionality of hyperspectral data. The experimental results have demonstrated excellent outcomes, effectively enhancing the efficiency of image processing.

Convolutional neural network (CNN) is the most frequently employed deep learning model that performs excellent classification effect in feature extraction of hyperspectral data; it can be used to solve the problem of plastic film in seed cotton [16,17]. LeNet, AlexNet, and VGGNet are frequently employed neural network models in CNN which achieve high classification and recognition accuracy with great fusion with hyperspectral images. Hüseyin Fırat et al. proposed a method to effectively classify hyperspectral remote sensing images (HRSIs) based on PCA dimension reduction and LeNet-5 of the 3D-CNN model. The results showed that a 100% recognition and classification effect was obtained in all experimental data [18]. Jiang et al. obtained hyperspectral images of different types of pesticide residues and used the fusion of the AlexNet-CNN deep learning network to detect post-harvest pesticide residues in apples. The test results showed that when the number of training epochs was 10, the detection accuracy was 99.09% [19]. Zhao et al. recorded the waterlogging of cotton after seeding with hyperspectral images. Based on the comparison experiment of GoogLeNet Inception-v3 (GLNI-v3) and VGG-16 conducted by CNN, the classification accuracy of VGG-16 was 97.00% higher than that of GLNI-v3, and the method could provide theoretical support for the evaluation of cotton loss after waterlogging [20]. The aforementioned literature demonstrates that the CNN-based models (LeNet, AlexNet, VGGNet) mentioned above exhibit strong generalization and adaptive capabilities in processing hyperspectral images, resulting in effective application outcomes.

The combination of hyperspectral imaging and CNN techniques is commonly applied to the classification of remote-sensing images. However, there have been few reports of methods to identify the residual film in seed cotton. The academic paper presents a novel approach for removing film in seed cotton, which combines hyperspectral images and deep learning algorithm. The innovations are listed as follows:

(1) The study establishes an optimal method for dimensionality reduction of hyperspectral data, which can reduce redundant hyperspectral characteristic information and reduce the time and cost of subsequent neural network training.

(2) The study integrates hyperspectral imaging technology and deep learning algorithm to obtain the optimal AlexNet-PCA-12 model which can effectively remove the colorless and transparent film in seed cotton in the practical application.

The remainder of the paper is structured as follows. Section 2 describes the hyperspectral sorting system, the theory of dimensionality reduction and CNN. Section 3 illustrates the discussion of the results of dimension reduction and CNN experiments. Conclusions and viewpoints are provided in Section 4.

## 2. Materials and Methods

### 2.1. Hyperspectral Sorting System

#### 2.1.1. Experimental Materials

Gaia Sorter-Dual, a full-band hyperspectral Sorter, is used in conjunction with the hyperspectral camera “Image-λ-N25E-SWIR”. A total of 10 kg of machine-picked long-staple cotton from southern Xinjiang and 50 pieces of film of different sizes are picked out by skilled workers.

**Figure 1:**
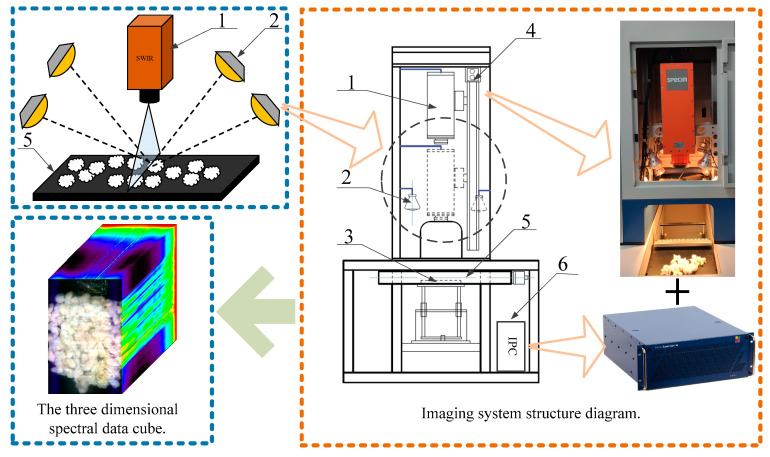
Hyperspectral imaging system. (1) Hyperspectral camera, (2) Halogen lamp, (3) Electronic control platform, (4) Distance regulating mechanism, (5) Transfer platform, (6) Industrial computer.

As shown in Figure 1, the hyperspectral imaging system can obtain the hyperspectral images of seed cotton mixed with the film: the resolution is 384 pixels × 600 pixels, the spectral range is 1000~2500 nm, 288 bands. The hyperspectral camera is positioned directly above the platform, with four halogen lamps symmetrically placed around it. The angle of irradiation of the halogen lamp can be adjusted arbitrarily. All halogen sources are adjusted to the position directly below the camera. The distance regulating mechanism is responsible for controlling the vertical motion of the hyperspectral camera in order to adjust the camera’s image surface. Additionally, the transfer platform can continuously move horizontally to capture continuous one-dimensional images. Since experimental subjects come in different sizes, the electronic control platform allows for vertical movement to create storage space for the subjects. The collected hyperspectral images can be regarded as 230,400 pieces of sample data, including 92,456 seed cotton samples, 63,478 film on cotton samples, 62,897 background samples and 11,569 film on background samples. The specific operation steps are as follows:

(1) The samples are placed on the transfer platform and irradiated uniform light from a halogen lamp. The reflected light is then captured by a hyperspectral camera, which provides one-dimensional spectral information.

(2) The transfer platform moves horizontally to obtain continuous one-dimensional spectral information, which is then transmitted to an industrial computer to generate hyperspectral images containing all the spectral information.

#### 2.1.2. Algorithm Environment

Hardware environment for Intel^®^Core (TM)i7-6700 CPU by Intel Semiconductor Co., Ltd. in Dalian, China, 16 GB RAM, and NVIDIA GeForce RTX 2080Ti by Taiwan Integrated Circuit Manufacturing Co., Ltd. in Taiwan, China, 11 GB was obtained. Software environment for tensorflow-gpu 2.0.0, spectral 0.22.1, sklearn 0.23.2, matplotib 3.2.2, kears 2.3.1, cuda 10.2.89, cudnn 7.6.5 was used employing the Python 3.6 programming language.

#### 2.1.3. Technical Route

**Figure 2:**
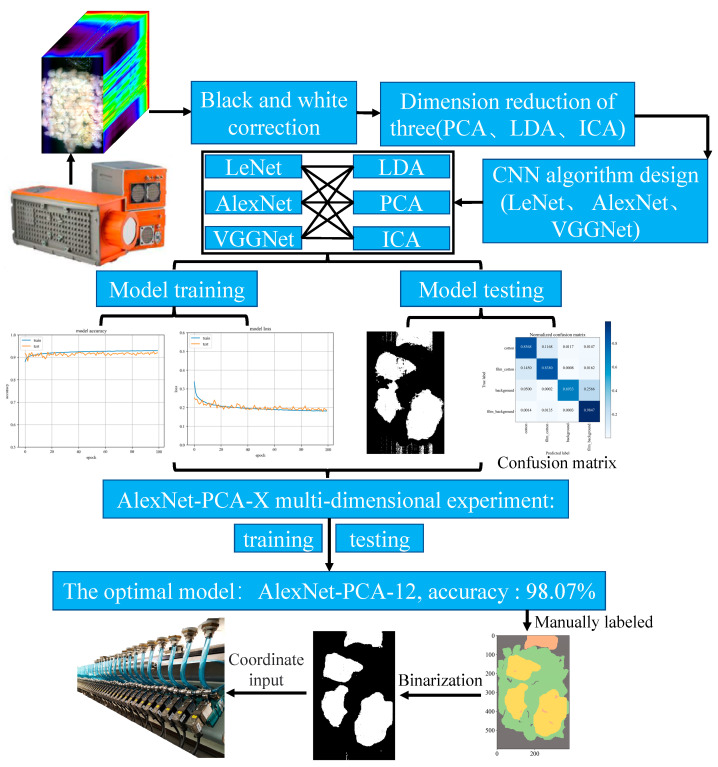
The technical route of the film sorting system.

The technical route of the film sorting system is illustrated in Figure 2, where seed cotton mixed with the film is given to the study subject. Firstly, a hyperspectral camera is used to collect 1000–2500 nm hyperspectral images. Secondly, the experimental validation involves nine models, including black and white correction, dimension reduction, and CNN training and testing. The purpose is to determine the best models for hyperspectral data dimension reduction and CNN. The binarization is established to display the recognition outcome of the optimal AlexNet-PCA-12 model, which has an eminent recognition accuracy of 98.07%. Finally, the coordinates of the film are fed into a high-speed spray valve to complete the film removal in the practical application sorting tests.

### 2.2. Black and White Correction of Hyperspectral Images

The stability of data can be impacted by environmental factors including light intensity and angular variations. In addition, there is a dark current in the camera and noise interference in the acquisition. Therefore, the hyperspectral images need to be corrected, which can remove ambient light interference and most of the noise in the image and effectively improve the classification and recognition accuracy of the subsequent model. The original hyperspectral images can be corrected by [20]

(1) $$I_{\mathrm{ref}}=\frac{I_{raw}-I_{dark}}{I_{white}-I_{dark}},$$

where $I_{ref}$ represents the corrected image, $I_{raw}$ is the original image, $I_{white}$ denotes standard correction image, $I_{dark}$ indicates background correction image.

### 2.3. Dimension Reduction of Hyperspectral Data

Dimension reduction can effectively eliminate noise and irrelevant information while also preventing data redundancy and dimension explosion caused by high-dimensional data during algorithmic processing [21]. At present, the dimension reduction methods of hyperspectral data mainly conclude linear discriminant analysis (LDA), principal component analysis (PCA), independent component analysis (ICA), etc. By extracting and mapping the main feature bands of the original data, these methods can effectively reduce the operating cost of the algorithm while ensuring the recognition accuracy of the algorithm.

#### 2.3.1. Linear Discriminant Analysis

LDA is a linear learning method that employs pattern recognition, machine learning, and other techniques to extract similar features of two objects or events from multiple datasets. These features are then combined to more accurately identify the differences between them [22].

Hyperspectral data contains an LDA multi-classification task, which needs to project the vector *x* of the *D* dimension to y of the *d* (*d* < *D*) dimension, and the projection equation can be provided by

(2) $$y=W^{T}x,$$

where *W* is the projection matrix and the projection direction of each column vector is perovided by $w_{i}$.

Multi-classification task data sets *X* can be written as

(3) $$X=\{x_{1}^{\left(1\right)},x_{2}^{\left(2\right)},\ldots ,x_{M_{1}}^{\left(1\right)},x_{1}^{\left(2\right)},\ldots x_{M_{N}}^{\left(N\right)}\},$$

where *N* represents the number of sample types, *i* indicates the kind of sample, $x_{j}^{\left(i\right)}$ denotes the *j* sample of class *i*, $M_{i}$ is the number of class *i* training samples (i=1,2,…,N).

The in-class divergence matrix $S_{w}$ is obtained as [22]

(4) $$S_{w}=\sum_{i=1}^{N}\sum_{j=1}^{M_{i}}p\left(i,j\right)\left(x_{j}^{\left(i\right)}-\mu_{i}\right){\left(x_{j}^{\left(i\right)}-\mu_{i}\right)}^{T},$$

where $\mu_{i}$ presents the mean of training samples of class *i*, p(i,j) is the probability of $x_{j}^{\left(i\right)}$.

The overall divergence matrix $S_{t}$ is given by [22]

(5) $$S_{t}=\sum_{i=1}^{N}\sum_{j=1}^{M_{i}}p\left(i,j\right)\left(x_{j}^{\left(i\right)}-\mu \right){\left(x_{j}^{\left(i\right)}-\mu \right)}^{T},$$

where μ represents the mean value of all training samples.

Interclass divergence matrix $S_{b}$ [22] is

(6) $$\begin{array}{ll} S_{b} & =S_{t}-S_{w}\\ & =\sum_{i=1}^{N}p\left(i\right)\left(\mu_{i}-\mu \right){\left(\mu_{i}-\mu \right)}^{T} \end{array},$$

where p(i) denotes the probability of class *i*.

Then, we obtain the objective function *J* [22]:

(7) $$\begin{array}{l} J=\frac{W^{T}S_{b}W}{W^{T}S_{w}W}\\ S_{W}^{-1}S_{b}W=\lambda W \end{array}.$$

The projection matrix *W* of the *d* dimension can be obtained by calculating the largest *d* eigenvalues of $S_{W}^{-1}S_{b}$ and the corresponding *d* eigenvectors; *d* (*d* < *N*) is the dimension after dimensionality reduction of hyperspectral data.

#### 2.3.2. Principal Component Analysis

PCA is a dimension reduction algorithm based on the discrete Karhunen–Loeve transform for extracting the main feature components of multivariate data information [23]. Although the majority of the noise in the image can be removed using PCA, it has greater advantages in terms of time complexity.

**Data conversion.** While reading is performed in the hyperspectral image data, each band data is converted into a one-dimensional vector. The hyperspectral image data are assumed to have a total of *N* bands with a w×h resolution, which can be represented as a matrix of (w×h)×N. Here, the band *i* can be expressed as

(8) $$x^{i}=\left[x_{1}^{i},x_{2}^{i},\ldots ,x_{w\times h}^{i}\right],\left(i=1,2\ldots N\right).$$

**For the eigenspace.** The mean vector of all bands is calculated as [24]

(9) $$\bar{x}=\frac{1}{N}\sum_{i}^{N}x^{i}.$$

The distance vector between each band and the average band can be obtained as

(10) $$d_{i}=x^{i}-\bar{x}.$$

We set the matrix *B* as

(11) $$B=\left[d_{1},d_{2},\ldots ,d_{N}\right].$$

Then, the covariance matrix can be obtained as follows [24]:

(12) $$\frac{1}{N}BB^{T}=\frac{1}{N}\sum_{i}^{N}d_{i}d_{i}^{T}.$$

The transpose matrix in Formula (12) can be written as

(13) $${\left(BB^{T}\right)}^{T}=B^{T}B.$$

Since Formula (12) is a high-dimensional vector of (w×h)×(w×h), the calculation of eigenvectors of the first Z(Z≤N) large eigenvalues of the covariance matrix is too large, while Formula (13) is a low-dimensional vector of N×N, and therefore its eigenvalue can be calculated first [25]:

(14) $$v_{j}=Bu_{j}\lambda_{j}^{-\frac{1}{2}},\;\left(j=1,2,\ldots ,Z\right),$$

where $\lambda_{j}$ presents the eigenvalue of Formula (12) and $u_{j}$ is the eigenvector of Formula (13). The eigenspace $v_{j}$ can be formed by the eigenvalues of Formula (13):

(15) $$W=\left\{v_{1},v_{2},\ldots ,v_{Z}\right\}.$$

**Projection and similarity detection.** The difference vector between each band and the average band is projected into the eigenspace, and the eigenvector *i* is expressed as

(16) $$P_{i}=W^{T}d_{i},\;\left(i=1,2,\ldots ,N\right).$$

The Euclidean distance is written as [25]

(17) $$\varepsilon_{i}={\left\|P_{i}-P_{k}\right\|}^{2},\;\left(i,k=1,2,\ldots N\right).$$

When using PCA dimension reduction, similarity between images is determined by the Euclidean distance. A smaller Euclidean distance indicates a greater similarity and better results. After this operation, *n* eigenvector *P* with minimum Euclidean distance is tested to form a fresh hyperspectral data set, where *n* < *N* is the dimension of hyperspectral data after dimensionality reduction.

#### 2.3.3. Independent Component Analysis

ICA is a method to find data intrinsic components from multi-dimensional statistical data which focuses on data analysis from independent sources, decomposing multivariate signals into different non-Gaussian signals [26]. Hyperspectral image data *X* can be regarded as a two-dimensional matrix with *N* rows and *L* columns (L=w×h). Hyperspectral data with band *n* (*n* < *N*) can be obtained through ICA to achieve the purpose of dimension reduction.

ICA of *X* can be expressed as [15]

(18) $$X=AS=\sum_{d=1}^{N}a_{d}s_{d},$$

where *N* is the number of bands. *d* denotes band index number (d=1,2,…,N), $A=\left(a_{1},a_{2},\ldots ,a_{d},\ldots ,a_{N}\right)$ presents a mixing matrix, $a_{d}={\left(a_{1d},a_{2d},\ldots ,a_{Nd}\right)}^{T}$ denotes the column vector of *A*, $S={\left(s_{1},s_{2},\ldots ,s_{d},\ldots s_{N}\right)}^{T}$ indicates an independent component matrix, $s_{d}={\left(s_{d1},s_{d2},\ldots ,a_{dN}\right)}^{T}$ is the row vector of *S*.

We set $W=A^{-1}$ according to Formula (18) [15]:

(19) $$S=A^{-1}X=WX,$$

where $\left(w_{1},w_{2},\ldots w_{d},\ldots ,w_{N}\right)$ is defined as the transformation matrix *W*, ${\left(w_{1d},w_{2d},\ldots ,w_{Nd}\right)}^{T}$ presents the column vector of *W*. The independent component *S* is obtained by finding the appropriate transformation matrix *W* for the independent statistical and non-Gaussian properties of each component according to the principle of the central limit theorem.

Depending on the choice of the objective function, ICA includes FastICA, Projection pursuit, and Infomax, which mainly extract independent components by increasing non-Gaussian properties, reducing mutual information, and performing maximum likelihood estimation [15]. The FastICA approach which adopts batch processing to incorporate a huge quantity of sample data into the iterative process is utilized to optimize independent components. It also establishes negative entropy as a non-Gaussian measure of random variables. The steps of solving independent components by the FastICA algorithm can be described as follows:

**(1) Bleaching data.** We set the average value of hyperspectral image data *X* as $\bar{X}$ and perform decentralized processing on the data to obtain

(20) $$P=X-\bar{X}.$$

The covariance matrix for *P* can be written as [27]

(21) $$C=\mathrm{cov}\left(P,P^{T}\right).$$

The eigenvalue λ and eigenvalue diagonal matrix *D* are calculated through |λI−C|=0 where *I* denotes the unit vector; the eigenvector *E* can be found by (λI−C)E=0.

There is a bleaching transformation matrix $U=D^{{-}_{2}^{1}}E^{T}$, and the data after bleaching are obtained as [27]

(22) Z=U×P.

**(2) Finding the matrix *W*.** We let *k* be the number of iterations, and the iterative computation of $w^{\left(k\right)}$ can be expressed as [27]

(23) $$w^{\left(k\right)}=E\left\{ZG\left(w_{d}^{{\left(k-1\right)}^{T}}Z\right)\right\}-E\left\{Zg\left(w_{d}^{{\left(k-1\right)}^{T}}Z\right)\right\}\times w_{d}^{\left(k-1\right)},$$

where $G\left(t\right)=\tanh\left(t\right)=\left(e^{t}-e^{-t}\right)/\left(e^{t}+e^{-t}\right)$ denotes a hyperbolic tangent function, g(t) is the first derivative of G(t), E(•) indicates mean function.

We orthogonalize and standardize the matrix *W* [27]:

(24) $$\begin{array}{l} \sum_{j=1}^{d-1}\left(w_{d}^{{\left(k\right)}^{T}}w_{j}\right)w_{j}\to w_{d}^{\left(k\right)}\\ \frac{w_{d}^{\left(k\right)}}{\left\|w_{d}^{\left(k\right)}\right\|}\to w_{d}^{\left(k\right)} \end{array}.$$

For any real number ε greater than *0*, if $\left|w_{d}^{{\left(k\right)}^{T}}w_{d}^{\left(k-1\right)}-1\right|<\varepsilon$, the $w_{d}$ converges; otherwise, k=k+1 takes Formula (23) to continue the iteration. The column vector $w_{d}$ of *W* can be obtained from Formula (24), and *d* (d=1,2,…,N) is the index number of each band.

When d=N, the matrix *W* is calculated as follows:

(25) $$W=\left(w_{1},w_{2},\ldots w_{d},\ldots ,w_{N}\right).$$

By taking the matrix *W* into Formula (19), the independent component *S* can be solved.

**(3) Selecting band.** The matrix *W* is defined as $\left(w_{1},w_{2},\ldots w_{j},\ldots ,w_{N}\right)$, the column vector *d* of *W* is defined as ${\left(w_{1j},w_{2j},\ldots w_{ij},\ldots ,w_{Nj}\right)}^{T}(i,j=1,2,\ldots N)$, where $w_{ij}$ indicates the capacity of the *j* band containing *i* independent component information. By calculating the average absolute weight factor, it can assess how much of each band contains independent component information:

(26) $$\bar{w_{j}}=\frac{1}{N}\sum_{i=1}^{N}\left|w_{ij}\right|.$$

The gained *n* bands with the largest average weight coefficient $\bar{w_{j}}$ are formed into a new low-dimensional image to achieve the dimensionality reduction of the hyperspectral image; *n* (*n* < *N*) is the number of bands after the dimensionality reduction of the hyperspectral data.

### 2.4. Construction of the Convolutional Neural Network

The convolutional neural network mainly concludes with an input layer, convolution layer, pooling layer, fully connected layer, and output layer, which can effectively solve the over-fitting problem [28]. The research illustrates a 2D-CNN-based method for hyperspectral image classification which can reduce the training cost while ensuring high classification and recognition accuracy.

**Convolution layer.** The convolution layer applies a convolution kernel to transform the input matrix into a unit matrix for the next layer. During forward propagation, the convolution kernel computes the nodes in the right unit matrix by using the nodes in the left input matrix [29]. Multiple convolution kernels are used to convolve with input image data, and a series of feature graphs are obtained through an activation function after biasing [30]. In the paper, the ReLU activation function is utilized to map the input of neurons to the output; its nonlinear characteristics are introduced into the neural network, enabling its application to various nonlinear models. The convolution formula is expressed as follows [29]:

(27) $$X_{j}^{l}=f\left(\sum_{i\in M_{j}}X_{i}^{l-1}•w_{ij}^{l}+b_{j}^{l}\right),$$

where $X_{j}^{l}$ denotes the j element of the *l* layer, $M_{j}$ stands for j convolution area of the l−1 layer feature map, $X_{i}^{l-1}$ presents the elements, $w_{ij}^{l}$ is the weight of the corresponding convolutional kernel matrix, $b_{j}^{l}$ is the offset item, f(⋅) indicates the activation function, $\sum_{i\in M_{j}}X_{i}^{l-1}•w_{ij}^{l}$ is the convolution formula.

**Pooling layer.** If all the features obtained through convolution are inputted into the classifier, a significant amount of computation is required to handle it. In this case, the Pooling function is required to process the feature maps obtained by convolution, and the Max pooling method is utilized in this paper. The pooled element matrix can reduce the dimension of the feature information obtained from the convolution layer and reduce the size of the matrix in the direction of height and width while ensuring the invariance of the feature scale. Meanwhile, the number of parameters of the whole neural network can be reduced, thus improving the generalization ability of the model [31].

**Fully connected layer.** With multi-layer convolution and pooling processing, images are gradually extracted with higher-level and more abstract feature information, which is classified by fully connected layers [32]. After unrolling the input feature vector into one dimension, the fully connected layer outputs the result via weighted summation and activation functions. The output formula is [29]

(28) $$y^{k}=f\left(w^{k}x^{k-1}+b^{k}\right),$$

where *k* is the serial number of the network layer, $y^{k}$ is the output, $x^{k-1}$ represents the expanded one-dimensional eigenvector, $w^{k}$ stands for the weight coefficient, $b^{k}$ is the offset item. f(⋅) is a model for probabilistic computation and an activation function suitable for classification tasks, which can be formulated as follows:

(29) $$y=\mathrm{softmax}(w_{ij}x_{j}+b).$$

The softmax loss function is structured in the fully connected layer to measure the solving accuracy of the problem, and loss function is adopted to describe the degree of dissatisfaction with the classification result. The effect of the neural network model is defined by the loss function. The tinier the loss value, the tinier the deviation between the result obtained by the model and the real value [33].

The purpose of neural network optimization is to accurately and timely update the parameters. Two optimization methods are employed for neural networks in the paper: the first step is the Gradient descent algorithm, and the second is the Back propagation algorithm. The optimization method of Gradient descent is to randomly select a function on the training data during the iteration process, which ensures the rapid update of parameters in each iteration. The back propagation algorithm based on the gradient descent algorithm can not only calculate vector gradients, but also calculate multidimensional tensors [34].

To avoid training overfitting, the Dropout function is used in the fully connected layers to make the output of neurons in the hidden layer drop to zero with a certain percentage probability. Dropout disables some hidden layer nodes that do not participate in the forward propagation process of the CNN. Due to the stochastic nature of the Dropout, each sample input to the network corresponds to a different network structure, but all these structures share weight. Since a neuron cannot depend on additional specific neurons, it reduces the complexity of inter-neuron adaptation and enables them to learn deeper features [35].

### 2.5. Design of Intelligent Recognition Algorithm for Film in Seed Cotton

**Figure 3:**
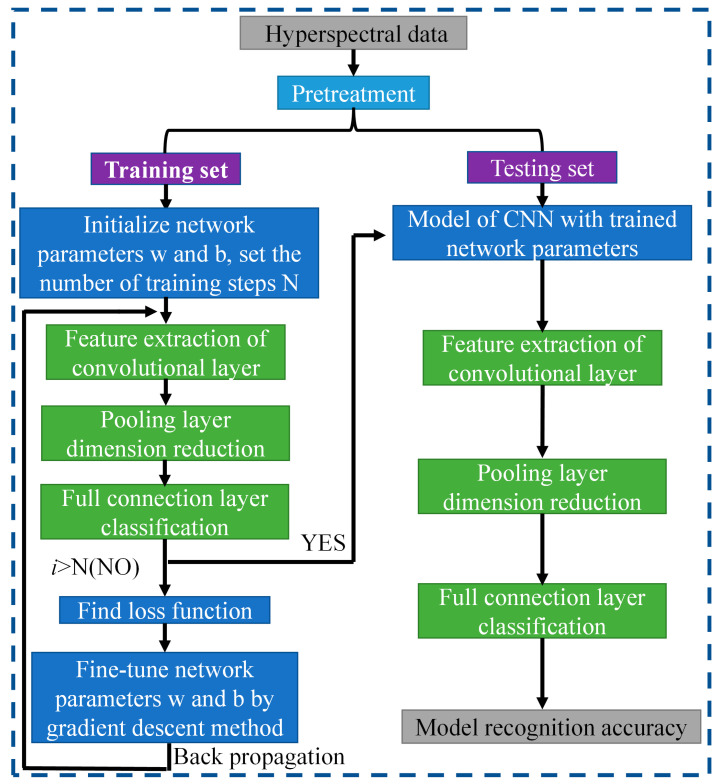
CNN schematic chart.

In this section, three CNN models based on LeNet, AlexNet, and VGGNet are constructed for hyperspectral image recognition. The CNN schematic is shown in Figure 3. The schematic involves two steps. Firstly, the hyperspectral data are used to train the model and extract useful image features. Secondly, the trained features are applied to the testing set for verification, and the resulting recognition accuracy is outputted. Additionally, the network parameters are regulated through gradient descent and back propagation algorithms, which ascertain network parameters in time.

To achieve optimal recognition results for hyperspectral image recognition, LeNet, AlexNet, and VGGNet models are altered accordingly. The specific parameters for each model are outlined in Table 1, Table 2 and Table 3. For facilitating CNN to input hyperspectral data and output recognition accuracy, CNN is set corresponding to the input layer and output layer, specifically as follows: In the input layer, 5 × 5 indicates the data size of the input convolutional network by manual division and D denotes the data dimension obtained after adopting different dimensionality reduction algorithms. In the output layer, the Softmax loss function outputs the probabilities of four units, which include “cotton”, “film on cotton”, “background”, and “film on background”.

## 3. Results and Discussion

### 3.1. Design of Intelligent Recognition Algorithm for Film in Seed Cotton

**Figure 4:**
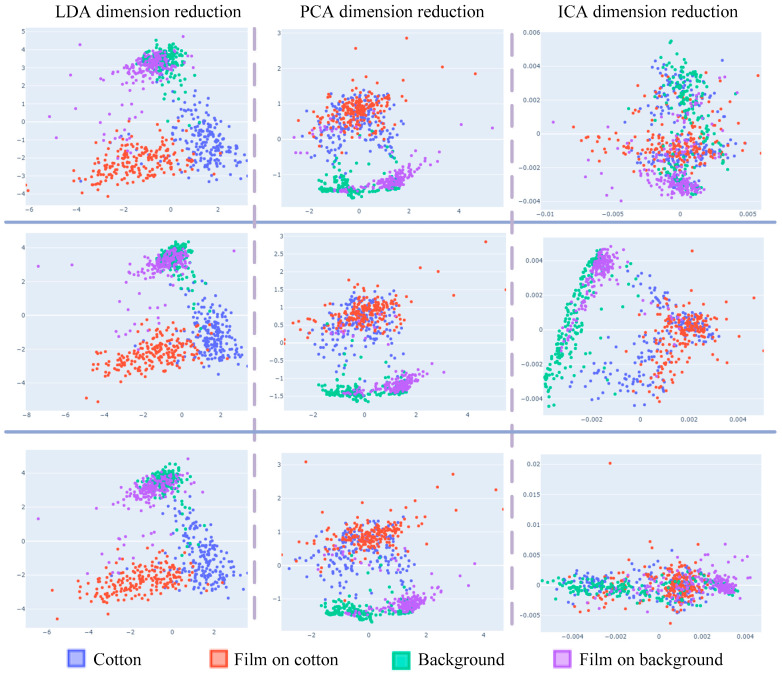
The scatter plot of three-dimensional reduction experiments.

To verify the generalization of the dimensional reduction, scatter plots are presented in Figure 4. The plots depict the application of different dimensional reduction methods on the same hyperspectral data from the three-dimensional reduction experiments. The scatter plots of dimensionality reduction for different batches of the same data under the same experimental conditions can be concluded as follows:

(1) Considering only the first two samples, LDA data have obvious clustering and separability, but LDA data cannot classify sample “background” accurately.

(2) ICA data classified the four types of samples differently in different batches, so it is not general to data from different batches of dimensionality reduction, and the trained model cannot achieve ideal results on the test.

(3) Considering only the first two samples, PCA dimension reduction has distinct aggregation and separability on “background” and “film on background”, while the data coincidence of the two samples “cotton” and “film on cotton” has no classification.

(4) The result shows that LDA has outstanding classification results with a dimensionality reduction of two for hyperspectral data. However, LDA can only reduce the data to three dimensions. Therefore, when the computer performance is satisfied, PCA obtains higher recognition accuracy when it is used to retain more dimensions.

### 3.2. CNN Model Training

Comparing three different dimension reduction methods (LDA, PCA, and ICA) after the hyperspectral data to a three-dimensional effect, three different structures are adopted CNN (LeNet, AlexNet, and VGGNet) for training and testing accuracy.

**Figure 5:**
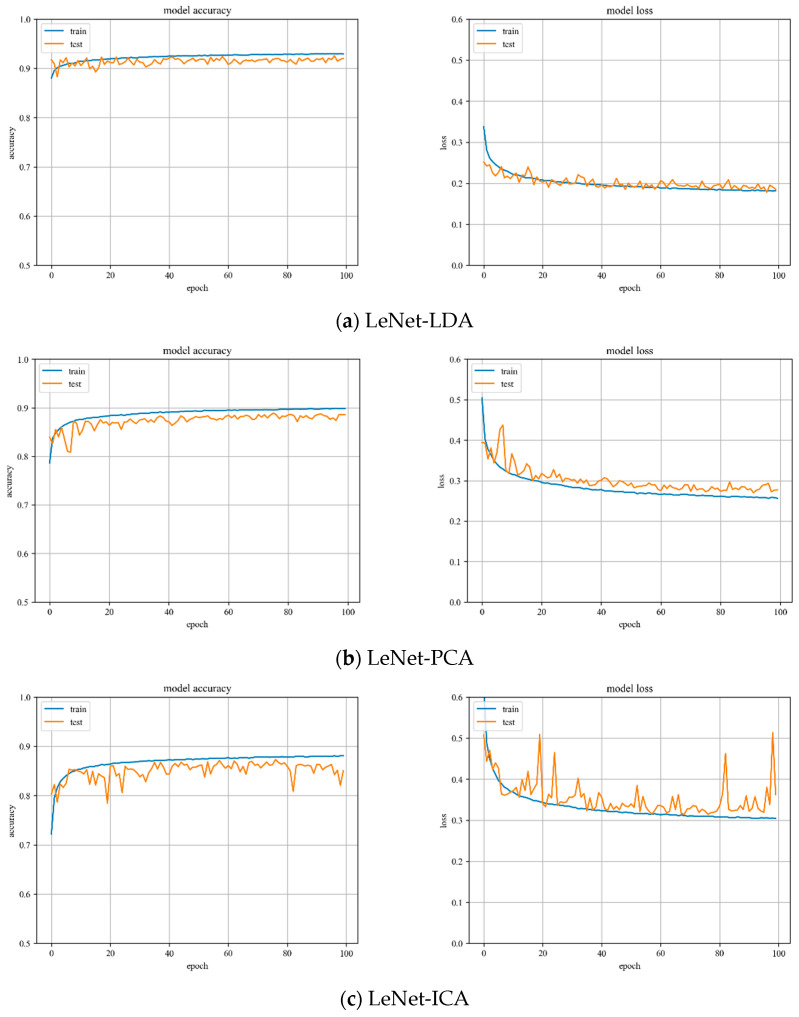
Accuracy curve and loss curve of LeNet model.

**LeNet model training.** Variations of training and testing accuracy of LeNet with the number of training epochs, training and testing loss curves are shown in Figure 5.

In Figure 5a, LDA recognition accuracy on the test set is about 92%, and the loss value is 0.15~0.2. In Figure 5b, PCA recognition accuracy on the test set is about 89%, and the loss value is 0.25~0.3. In Figure 5c, ICA recognition accuracy on the test set is about 85%, and the loss value is 0.3~0.35.

Despite the fact that LDA and PCA are relatively stable to changes throughout the training phase, PCA slightly underperforms the LeNet model with LDA hyperspectral data reduction. However, the LeNet model with ICA hyperspectral data reduction has the worst stability of the three.

**Figure 6:**
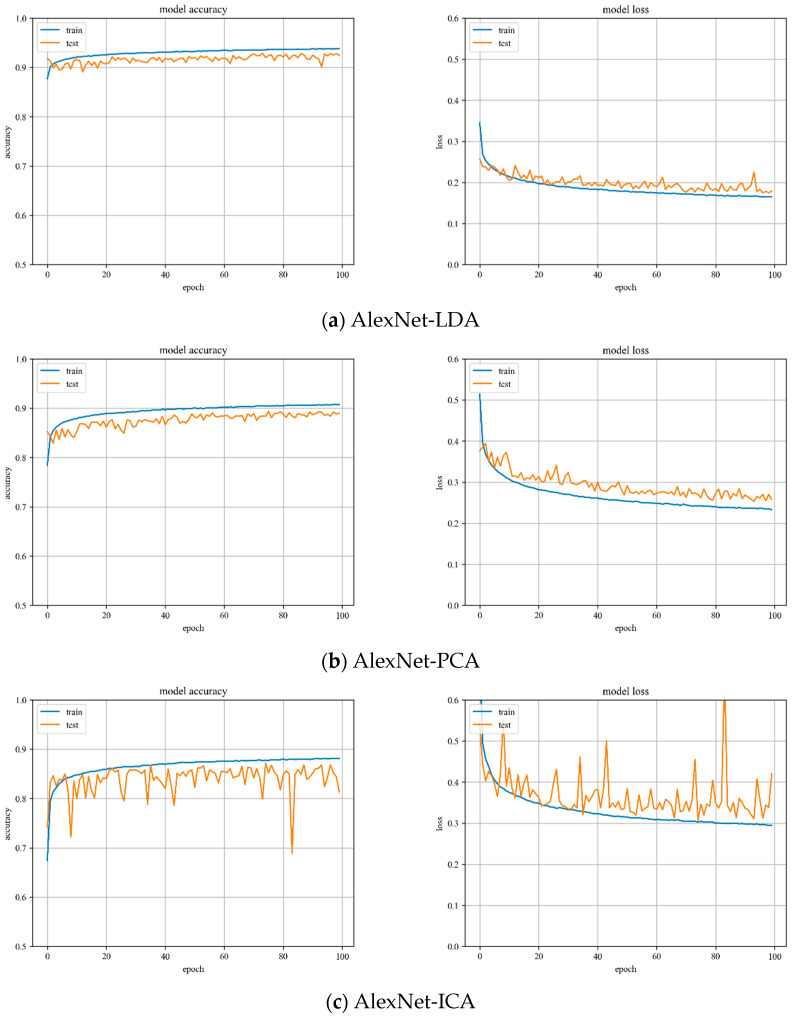
Accuracy curve and loss curve of AlexNet model.

**AlexNet model training.** Variations of training and testing accuracy of AlexNet with the number of training epochs, training and testing loss curves are shown in Figure 6.

In Figure 6a, LDA recognition accuracy on the test set is about 93%, and the loss value is 0.15~0.2. In Figure 6b, PCA recognition accuracy on the test set is about 90%, and the loss value is 0.25~0.3. In Figure 6c, ICA recognition accuracy on the test set is about 88%, and the loss value is 0.3~0.35.

Despite the fact that LDA and PCA are relatively stable to changes throughout the training phase, PCA slightly underperforms the AlexNet model with LDA hyperspectral data reduction. However, the AlexNet model with ICA hyperspectral data reduction has the worst stability of the three.

**Figure 7:**
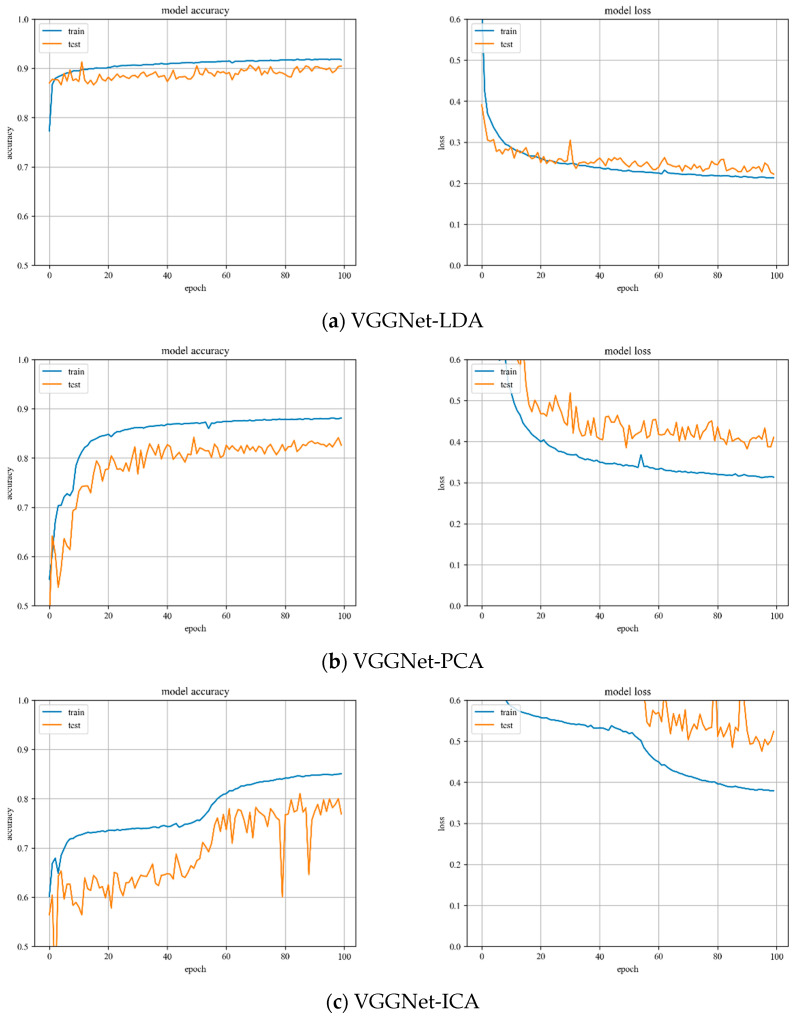
Accuracy curve and loss curve of VGGNet model.

**VGGNet model training.** Variation of training and testing accuracy of VGGNet with the number of training epochs, training and testing loss curves are shown in Figure 7.

In Figure 7a, LDA recognition accuracy on the test set is about 90%, and the loss value is 0.2~0.25. The LDA model is relatively stable to changes throughout the training process and has excellent model stability.

In Figure 7b, PCA recognition accuracy on the test set is about 84%, and the loss value is about 0.4. In Figure 7c, ICA recognition accuracy on the test set is about 80%, and the loss value fluctuates widely. Both models have minor stability during the training phase. The ICA results are inferior than the VGGNet model with PCA hyperspectral data reduction.

### 3.3. CNN Model Testing

The confusion matrix for the test samples for the different algorithmic models are illustrated in Table 4, Table 5 and Table 6. It can be seen that 1 is the cotton, 2 represents the film on cotton, 3 indicates the background, and 4 denotes the film on background, the diagonal expresses the probability of correct classification. The experimental data analysis is as follows:

(1) Using LDA and PCA dimensionality reduction hyperspectral data, it can be determined that the three kinds of CNN models have higher recognition accuracy for test samples. However, there are some errors in the classification of film samples on cotton and film samples on background, which is consistent with the conclusion of the scatter plots above.

(2) Since the hyperspectral data for ICA dimension reduction is not the same batch as the data during training, the extracted dimension information is unstable and the recognition effect is confused. Therefore, it cannot be applied to hyperspectral image recognition, which is consistent with the conclusion of the scatter plots above.

The Overall Accuracy (OA) of the test samples is illustrated in Table 7, representing the percentage of all samples that are accurately predicted. The results can be summarized as follows:

(1) When the hyperspectral data are reduced to three dimensions, the average OA of LDA is 91.68%, while PCA has an average OA of 87.08%; on the other hand, ICA has a lower average OA of 40.35%. Based on these results, it can be concluded that LDA demonstrates superior performance in terms of dimensionality reduction.

(2) The data in the table show that the CNN-based AlexNet model can achieve excellent recognition effects when the data are dimensionally reduced.

(3) When the dimension reduction of ICA is 3, it exhibits poor performance in terms of average OA compared to the other two dimensionality reduction methods. However, PCA can retain more dimension information to improve the recognition accuracy, which has more potential in practical applications.

**Figure 8:**
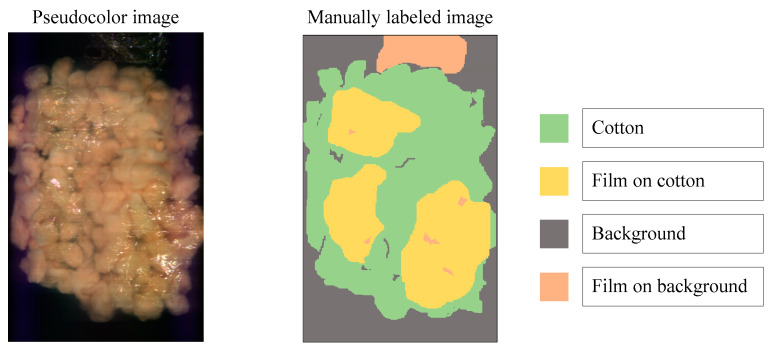
Pseudocolor and manually labeled images.

To distinguish the classification effects more intuitively, three bands are selected to display the hyperspectral data as pseudocolor images. Additionally, the spectral toolkit is used in the model tests to plot the predictions in the form of a two-dimensional image. The pseudocolor and manually labeled images are shown in Figure 8.

**Figure 9:**
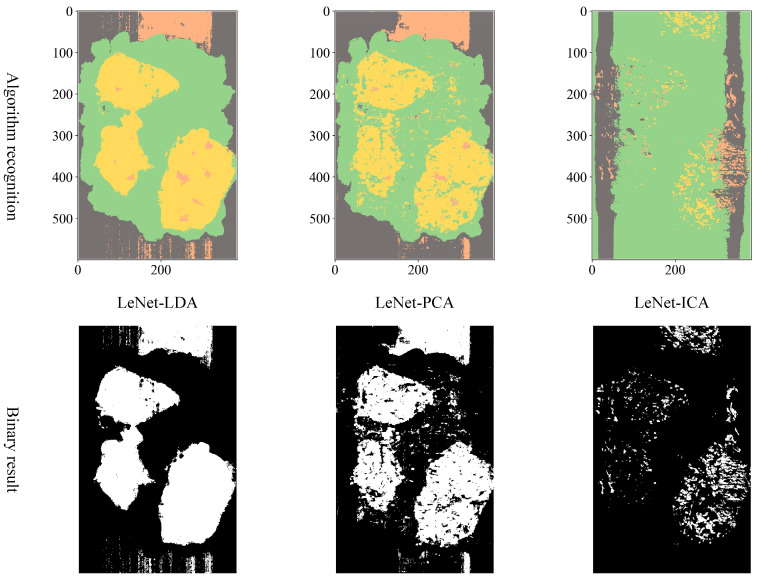
LeNet binarization images.

**Figure 10:**
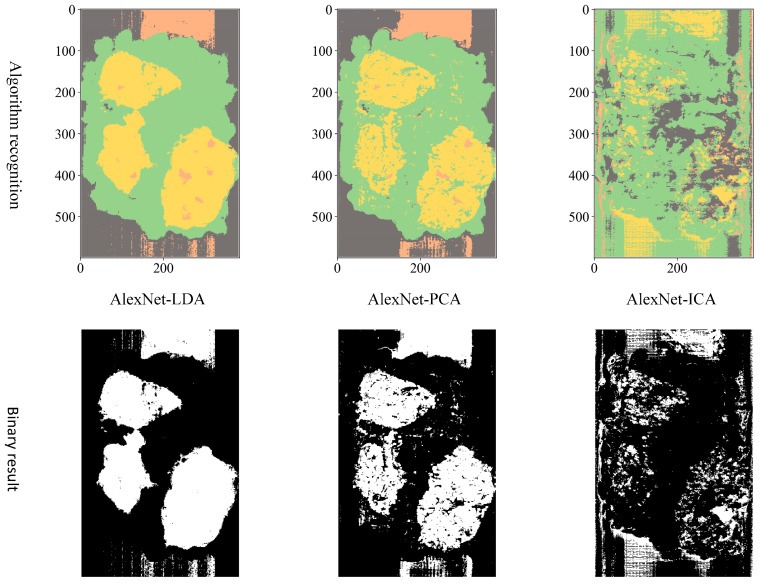
AlexNet binarization images.

**Figure 11:**
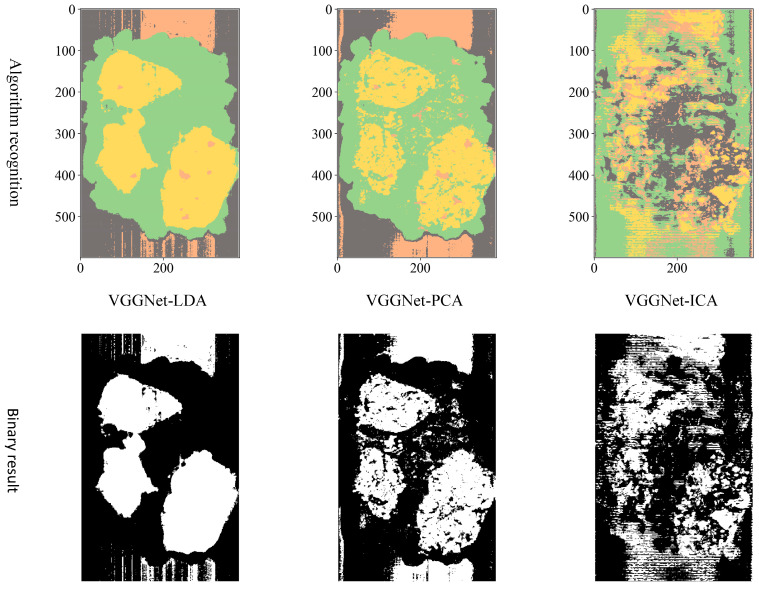
VGGNet binarization images.

Considering the actual sorting system only needed to locate the spatial coordinate position of the film, the classification results are combined from four categories into two categories: “film on cotton” and “film on background” are classified as film, and “cotton” and “background” are classified as non-film. The binarized images are shown in Figure 9, Figure 10 and Figure 11. It can be seen that the combination of the AlexNet neural network structure and the LDA algorithm indicate the best recognition results, while the VGGNet neural network structure and the ICA algorithm denote the worst recognition results.

Regarding the reduction to three dimensions, the above experiments validate the classification effect of different dimensionality reduction methods on hyperspectral data. The results show that LDA achieves the highest performance in terms of aggregation and separability of features preserved by dimensionality reduction of hyperspectral data. With limited device conditions for hyperspectral images, it is advisable to opt for LDA dimension reduction. However, due to the limitations of the LDA algorithm, the data can only be reduced to three dimensions. Therefore, when the computer performance meets the requirements, PCA achieves higher recognition accuracy when more dimensions are retained. In summary, the AlexNet-PCA multi-dimensional algorithm is experimented with to obtain the highest recognition accuracy for seed cotton mixed with the film.

### 3.4. AlexNet-PCA Multi-Dimensional Algorithm Experiment

#### 3.4.1. AlexNet-PCA Model Training

**Figure 12:**
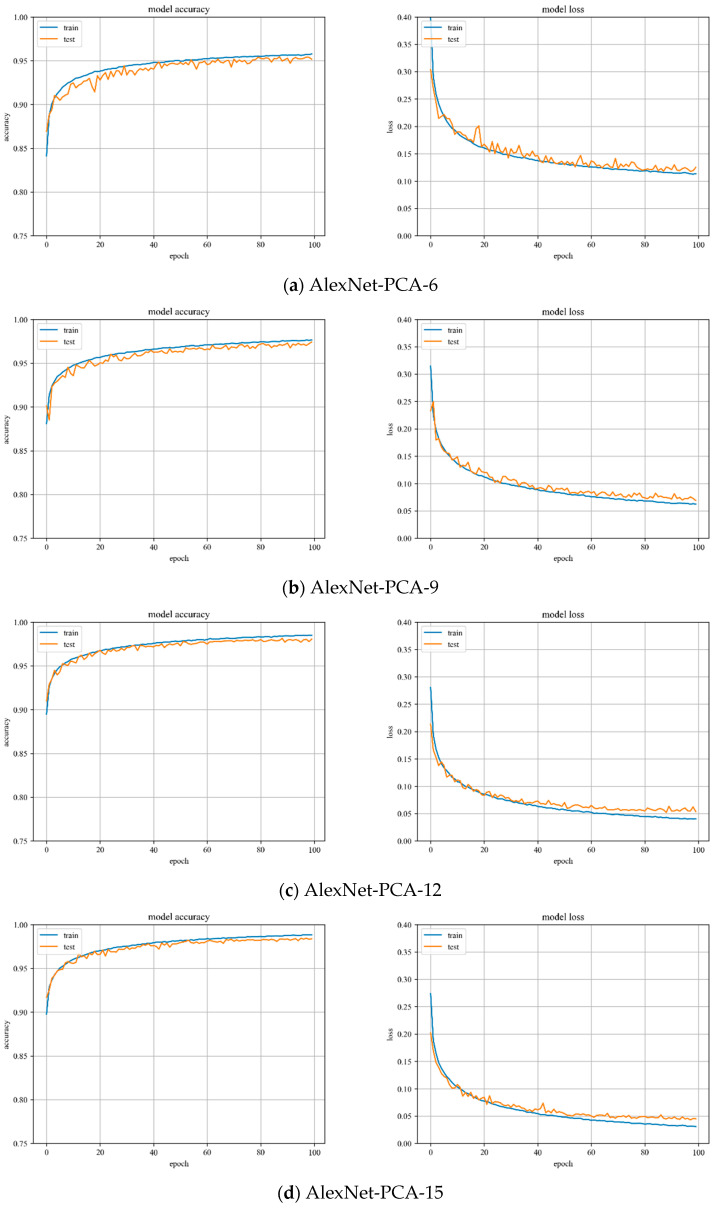
Accuracy curve and loss curve of AlexNet-PCA-X model.

In Figure 12, the accuracy and loss value curves of the AlexNet model are shown when PCA is used to reduce the dimensionality by 6, 9, 12, and 15. The accuracy curve of the test set starts to converge at the training process of up to 40 iterations and mostly peaks at the training process of up to 60 iterations. The variation is stable throughout the training process and the model has great stability.

#### 3.4.2. AlexNet-PCA Model Testing

As shown in Table 8, the experimental data analysis can be summarized as follows:

(1) The AlexNet-PCA algorithm for “cotton” and “background” has a minor number of errors in sample recognition classification. It can be attributed to the edge junction containing the reflection spectrum of both the cotton and the background.

(2) Misclassification is observed when using the AlexNet-PCA algorithm to identify the samples of “cotton” and “film on cotton”, “background” and “film on background”. It can be attributed to the weak reflection nature of the film, which leads to an indistinct discrimination of features.

Especially for the PCA dimension selection, a set of linearly increasing dimensions 3, 6, 9 and 12 is chosen for the AlexNet-PCA multi-dimensional algorithm experiment. The linearly increasing dimensions are conducive to the smooth change in the image curve between overall accuracy and dimension; hence, the experimental results are more intuitive.

**Figure 13:**
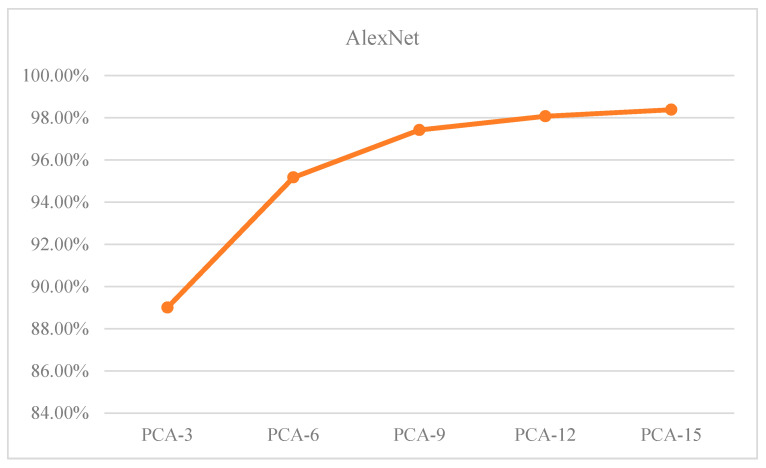
Relationship between OA and PCA.

The Overall Accuracy (OA) of the test sample is shown in Table 9, representing the percentage of all samples that are accurately predicted. As the number of dimensions retained by PCA increases, the OA of the samples keep increasing. Figure 13 illustrates the OA of the samples as a function of the dimensionality reduction of PCA. The data in Table 9 and Figure 13 show teh following:

(1) The increase in PCA dimensionality has an inverse relationship with the increase in accuracy.

(2) When the PCA dimension is set to 12, the proposed algorithm achieves a recognition accuracy of over 98%. Additionally, the overall classification accuracy of the samples begins to converge.

With the increase in PCA dimensionality reduction, the complexity of the neural network model also increases. However, the complexity of the model can lead to overfitting, which in turn can decrease the generalization ability of the model. In the study, we primarily utilize the Dropout method to avoid the overfitting problem. Dropout effectively weakens the connections between neuronal nodes, which reduces the network’s reliance on individual neurons and thereby enhances model generalization ability.

**Figure 14:**
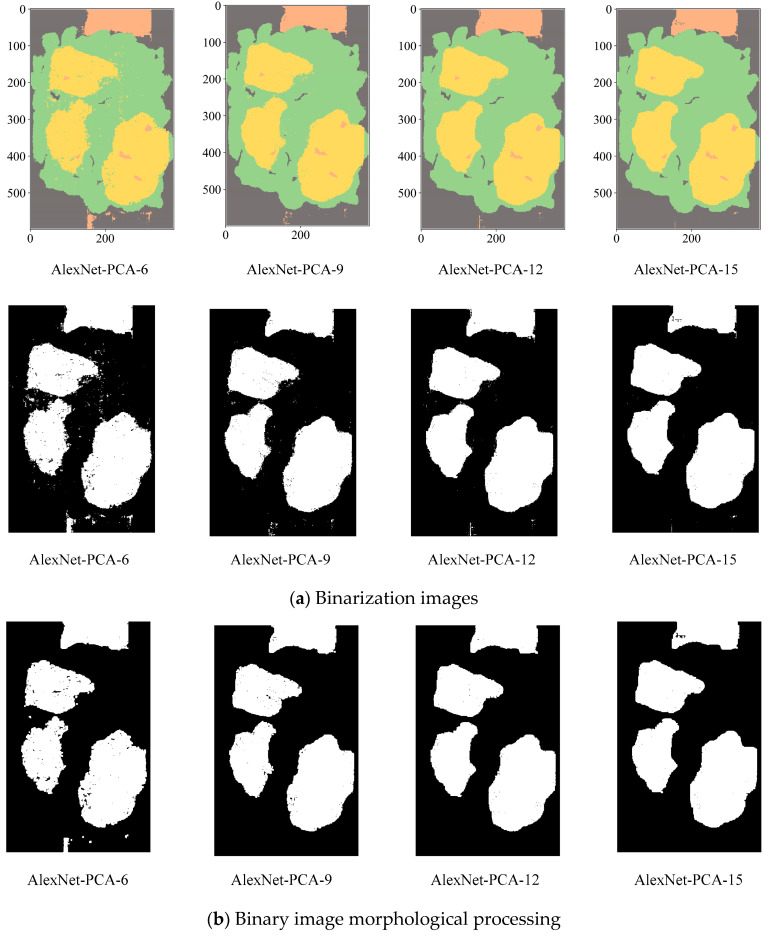
AlexNet-PCA-X binarization images.

The binarized images are shown in Figure 14a. As demonstrated in Figure 14b, the morphological method is utilized to perform an open operation on the binary image, which effectively minimizes the noise caused by light, dust, and artificial marks. As a result, the binary image contains the eliminated artifacts of identified small areas and image edges. The Figure 14 results show that:

(1) Despite reducing the dimensionality to six utilizing PCA, the post-processing results still exhibit significant imperfections. However, when the dimension reduction is increased to 12, the post-processed image results successfully meet the requirement of providing coordinates. With a dimension reduction of 15, there is no significant difference between the post-processed image results compared to those obtained with a dimension reduction of 12.

(2) Considering the relationship between speed of accuracy improvement, computer performance, image processing results, dimension reduction, and training cost, PCA with a dimension reduction of 12 is the optimal solution for computer performance.

#### 3.4.3. Practical Application Testing of Model AlexNet-PCA-12

**Figure 15:**
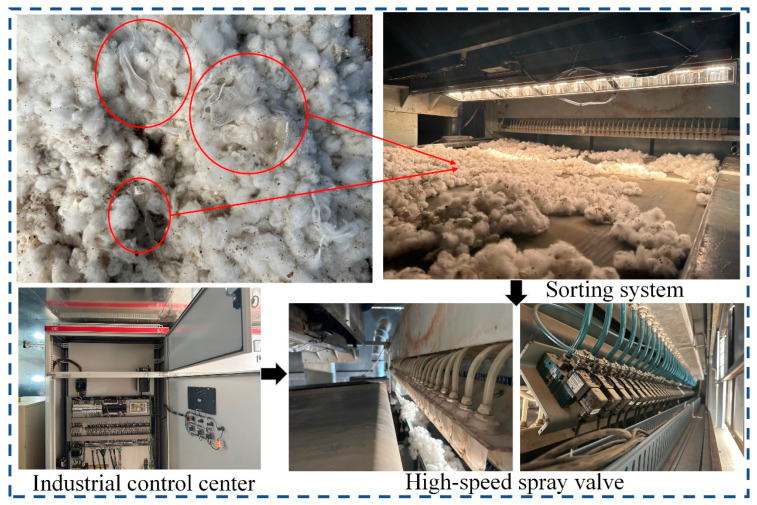
Hyperspectral sorting experiment.

As can be seen from the above, the AlexNet-PCA-12 model with the optimal recognition accuracy is obtained experimentally. To verify the feasibility of the research, an application sorting test of the algorithm is conducted in a cotton factory in Aksu, Xinjiang. As depicted in Figure 15, the computer platform running the algorithm obtains the actual coordinates of the film and inserts them into the industrial control center, which controls the response time of the high-speed spray valve to complete the film removal.

Table 10 shows the data of several sorting experiments: the overall removal rate of film is 97.02%, and the cotton sorting amount can reach 3.0 t/h, which meets the requirements of practical application.

### 3.5. Summary of Discussions and Results

This chapter focuses on three main tasks: collecting laboratory data, conducting tests on the algorithm, and comparing the visualized recognition results with the experimental results. The recognition effects of LeNet, AlexNet, and VGGNet neural networks combined with LDA, PCA, and ICA dimension reductions are compared and analyzed. Finally, the feasibility of the proposed optimal model is verified for practical applications.

## 4. Conclusions

Based on hyperspectral images and the deep learning intelligent recognition algorithm, a novel intelligent recognition method for seed cotton mixed with colorless and transparent film is proposed in this paper. The main research topics include the construction of hyperspectral classification systems, dimensionality reduction for hyperspectral data processing, construction of algorithmic recognition models, and the practical application sorting tests.

(1) The basic principles of hyperspectral imaging are studied and a hyperspectral classification system is designed for the intelligent classification of seed cotton mixed with the film. The system can obtain 288 hyperspectral data bands with a resolution of 384 pixels × 600 pixels and a spectral range of 1000~2500 nm, which provides an excellent data basis for the recognition of film in seed cotton.

(2) LDA, PCA, and ICA are utilized to reduce the dimension of hyperspectral data to settle the problems of high latitude, large amounts of data, and redundant information of hyperspectral data. Experimental results suggest that LDA and PCA generalized better than ICA. LDA is the best method when the dimensionality reduction is the same as the that of the three. PCA dimensionality reduction is more advantageous when computer performance is satisfied.

(3) An algorithm is successfully completed for hyperspectral image recognition of film in seed cotton. Based on the convolutional neural network architectures of LeNet, AlexNet, and VGGNet, the network model is constructed for hyperspectral image recognition applications in the seed cotton film domain.

(4) The combination test of the hyperspectral data dimension reduction algorithm (LDA, PCA, ICA) and the CNN model (LeNet, AlexNet, VGGNet) is completed. The experimental results illustrate that when the computer performance is satisfied, AlexNet-PCA-12 can achieve the best cost-to-performance ratio for both recognition and dimensionality reduction, and the recognition accuracy of the algorithm can reach 98.07%; the overall removal rate of film is 97.02% with the data of several sorting experiments in Aksu, Xinjiang.

On the whole, considering the influence of environmental factors such as light, humidity and dust in the practical application sorting tests, data under different environmental variables should be collected to further improve the generalization of the model. However, the research has potential applications in various fields, including but not limited to tea stalks removal, fruit and vegetable flaw separation, and pesticide residue detection in agricultural products. Further research can explore the use of photoelectric separation technology to enhance agricultural development.

## Figures and Tables

**Table 1:** LeNet structural parameter.

Type	Variables	Kernel Parameter	Data Output
Input layer	5 × 5 × D hyperspectral data set		
1-Conv	3 convolution kernels	Size: 3 × 3. All zero-filling. Step: 1	ReLU activation function
2-Pool	Max pooling	Size: 3 × 3. All zero-filling. Step: 2	
3-Conv	9 convolution kernels	Size: 3 × 3. All zero-filling. Step: 1	ReLU activation function
4-Pool	Max pooling	Size: 3 × 3. All zero-filling. Step: 2	Dropout drops 25% weight
Flatten layer	Convert multi-dimensional input into one dimension		
FC	Input neuron number: 108. Output neuron number: 18		
Output layer	Softmax loss function outputs the probabilities of four units.		

LeNet structure mainly consists of 2 Convs, 2 Pools, and 1 FC. “Conv” is convolution layer, “Pool” denotes pooling layer, “FC” indicates fully connected layer.

**Table 2:** AlexNet structural parameter.

Type	Variables	Kernel Parameter	Data Output
Input layer	5 × 5 × D hyperspectral data set		
1-Conv	3 convolution kernels	Size: 3 × 3. All zero-filling. Step: 1	ReLU activation function
1-Pool	Max pooling	Size: 3 × 3. All zero-filling. Step: 2	
2-Conv	9 convolution kernels	Size: 3 × 3. All zero-filling. Step: 1	ReLU activation function
2-Pool	Max pooling	Size: 3 × 3. All zero-filling. Step: 2	
3-Conv	12 convolution kernels	Size: 3 × 3. All zero-filling. Step: 1	ReLU activation function
4-Conv	12 convolution kernels	Size: 3 × 3. All zero-filling. Step: 1	ReLU activation function
5-Conv	9 convolution kernels	Size: 3 × 3. All zero-filling. Step: 1	ReLU activation function
5-Pool	Max pooling	Size: 3 × 3. All zero-filling. Step: 2	
Flatten layer	Convert multi-dimensional input into one dimension		
FC	Input neuron number: 27. Output neuron number: 60		
Output layer	Dropout drops 50% weight, Softmax loss function outputs the probabilities of four units.		

AlexNet structure mainly consists of 3 convolution groups (including 1 Conv and 1 Pool), 2 Convs, and 1 FC. “Conv” is convolution layer, “Pool” denotes pooling layer, “FC” indicates fully connected layer.

**Table 3:** VGGNet structural parameter.

Type	Variables	Kernel Parameter	Data Output
Input layer	5 × 5 × D hyperspectral data set		
1-Conv	3 convolution kernels	Size: 3 × 3. All zero-filling. Step: 1	ReLU activation function
1-Conv	3 convolution kernels	Size: 3 × 3. All zero-filling. Step: 1	ReLU activation function
2-Pool	Max pooling	Size: 2 × 2. All zero-filling. Step: 2	Dropout drops 20% weight
3-Conv	6 convolution kernels	Size: 3 × 3. All zero-filling. Step: 1	ReLU activation function
3-Conv	6 convolution kernels	Size: 3 × 3. All zero-filling. Step: 1	ReLU activation function
4-Pool	Max pooling	Size: 2 × 2. All zero-filling. Step: 2	Dropout drops 20% weight
5-Conv	12 convolution kernels	Size: 3 × 3. All zero-filling. Step: 1	ReLU activation function
5-Conv	12 convolution kernels	Size: 3 × 3. All zero-filling. Step: 1	ReLU activation function
5-Conv	12 convolution kernels	Size: 3 × 3. All zero-filling. Step: 1	ReLU activation function
6-Pool	Max pooling	Size: 2 × 2. All zero-filling. Step: 2	Dropout drops 20% weight
7-Conv	24 convolution kernels	Size: 3 × 3. All zero-filling. Step: 1	ReLU activation function
7-Conv	24 convolution kernels	Size: 3 × 3. All zero-filling. Step: 1	ReLU activation function
7-Conv	24 convolution kernels	Size: 3 × 3. All zero-filling. Step: 1	ReLU activation function
8-Pool	Max pooling	Size: 2 × 2. All zero-filling. Step: 2	Dropout drops 20% weight
9-Conv	24 convolution kernels	Size: 3 × 3. All zero-filling. Step: 1	ReLU activation function
9-Conv	24 convolution kernels	Size: 3 × 3. All zero-filling. Step: 1	ReLU activation function
9-Conv	24 convolution kernels	Size: 3 × 3. All zero-filling. Step: 1	ReLU activation function
10-Pool	Max pooling	Size: 2 × 2. All zero-filling. Step: 2	Dropout drops 20% weight
Flatten layer	Convert multi-dimensional input into one dimension		
FC	Input neuron number: 72. Output neuron number: 24		
Output layer	Dropout drops 20% weight, Softmax loss function outputs the probabilities of four units.		

VGGNet structure mainly consists of 5 convolution groups (including 2 or 3 Convs), 5 Pools, and 1 FC. “Conv” is convolution layer, “Pool” denotes pooling layer, “FC” indicates fully connected layer.

**Table 4:** LDA sample confusion matrix (%).

Model		Predictive	1	2	3	4
	Actual					
LeNet	1		91.89	5.72	2.22	0.17
	2		4.78	93.26	0.12	1.83
	3		3.99	0.05	90.49	5.47
	4		0.09	0.86	2.07	96.97
AlexNet	1		93.10	4.60	2.08	0.22
	2		5.17	92.87	0.18	1.77
	3		3.60	0.03	90.03	6.34
	4		0.00	0.81	1.24	97.95
VGGNet	1		90.44	6.84	2.48	0.23
	2		4.06	95.13	0.04	0.77
	3		4.40	0.07	84.55	10.98
	4		0.03	1.90	0.95	97.12

**Table 5:** PCA sample confusion matrix (%).

Model		Predictive	1	2	3	4
	Actual					
LeNet	1		89.13	8.86	1.57	0.44
	2		15.31	83.88	0.08	0.72
	3		3.83	0.08	91.15	4.94
	4		0.23	1.33	1.82	96.63
AlexNet	1		91.80	6.72	1.18	0.30
	2		14.98	84.41	0.08	0.53
	3		3.97	0.10	87.81	8.11
	4		0.09	1.35	0.17	98.39
VGGNet	1		87.95	8.75	2.11	1.19
	2		16.43	82.05	0.08	1.44
	3		4.11	0.02	72.40	23.47
	4		0.40	0.81	0.00	98.79

**Table 6:** ICA sample confusion matrix (%).

Model		Predictive	1	2	3	4
	Actual					
LeNet	1		75.24	0.07	23.07	1.62
	2		69.72	2.80	14.28	13.20
	3		90.51	0.75	8.62	0.12
	4		74.90	23.22	0.13	1.75
AlexNet	1		70.05	5.87	16.41	7.67
	2		32.78	32.72	15.84	18.65
	3		78.95	0.40	19.75	0.90
	4		65.08	24.49	5.19	5.25
VGGNet	1		48.65	18.68	21.23	11.44
	2		29.46	38.76	8.64	23.14
	3		28.10	10.37	51.14	10.39
	4		18.10	58.86	9.01	14.03

**Table 7:** Overall accuracy of the test samples (%).

	LDA	PCA	ICA
LeNet	92.14	88.61	33.41
AlexNet	92.45	89.00	42.78
VGGNet	90.46	83.63	44.86
Average	91.68	87.08	40.35

**Table 8:** AlexNet-PCA-X sample confusion matrix (%).

Model		Predictive	1	2	3	4
	Actual					
AlexNet-PCA-6	1		94.53	4.12	1.27	0.08
	2		3.18	96.21	0.11	0.49
	3		2.30	0.01	94.44	3.25
	4		0.03	0.95	0.29	98.73
AlexNet-PCA-9	1		96.88	1.67	1.37	0.07
	2		1.95	97.71	0.05	0.29
	3		1.06	0.03	97.68	1.23
	4		0.03	0.81	0.43	98.73
AlexNet-PCA-12	1		98.04	1.20	0.73	0.04
	2		1.00	98.75	0.02	0.23
	3		1.55	0.04	97.34	1.07
	4		0.03	0.92	0.43	98.62
AlexNet-PCA-15	1		98.38	0.50	1.11	0.01
	2		1.35	98.45	0.04	0.16
	3		0.96	0.01	98.38	0.66
	4		0.00	0.63	1.27	98.10

**Table 9:** Overall accuracy of AlexNet-PCA-X test samples (%).

Dimension	PCA-3	PCA-6	PCA-9	PCA-12	PCA-15
AlexNet	89.00	95.17	97.42	98.07	98.38

**Table 10:** Application of test results.

Quantity of Trials	Quantity of Films in Cotton	Quantity of Removal Films	Removal Accuracy
1	132	128	96.97%
2	112	109	97.32%
3	98	95	96.94%
4	146	142	97.26%
5	157	152	96.82%
6	104	100	96.15%
7	128	125	97.66%
8	168	163	97.02%
9	84	82	97.62%
10	113	109	96.46%
Sum	1242	1205	97.02%

## Data Availability

Not applicable.
